# Seasonal variation of serum KL-6 concentrations is greater in patients with hypersensitivity pneumonitis

**DOI:** 10.1186/1471-2466-14-129

**Published:** 2014-08-07

**Authors:** Hiroshi Ohnishi, Shintaro Miyamoto, Shigeo Kawase, Tetsuya Kubota, Akihito Yokoyama

**Affiliations:** 1 Department of Hematology and Respiratory Medicine, Kochi Medical School, Kochi University, Oko-cho, Kohasu, Nankoku, Kochi 783-8505, Japan

**Keywords:** Biomarker, Hypersensitivity Pneumonitis, Interstitial lung disease, KL-6, Seasonal variation

## Abstract

**Background:**

Serum KL-6 is a useful biomarker for the diagnosis of interstitial lung diseases (ILD). However, KL-6 has not been used to discriminate different types of ILD. Serum KL-6 concentrations can vary depending on antigen exposure levels in patients with hypersensitivity pneumonitis (HP); however, seasonal changes in serum KL-6 concentrations in ILD have not been determined. We hypothesized that seasonal variation of serum KL-6 is greater in HP than for the other ILD. The aim of this study was to determine seasonal variation of serum KL-6 concentrations in various ILD.

**Methods:**

Serum KL-6 concentrations in the summer season from June 1 to September 30 and the winter season from November 1 to February 28 were retrospectively analyzed in patients with idiopathic pulmonary fibrosis (IPF, n = 16), non-specific interstitial pneumonia (NSIP, n = 16), collagen vascular disease-associated interstitial pneumonia (CVD-IP, n = 33), house-related HP (House-HP, n = 9), bird-related HP (Bird-HP, n = 9), and combined pulmonary fibrosis and emphysema (CPFE, n = 13).

**Results:**

Bird-HP and House-HP showed greater seasonal serum KL-6 variation than the other ILD. Serum KL-6 concentrations in Bird-HP were significantly increased in the winter and KL-6 concentrations in House-HP were significantly increased in the summer. Serum KL-6 variation was significantly greater in acute HP than chronic HP. Receiver operating characteristic curve analysis revealed that greater seasonal variation in serum KL-6 concentrations is diagnostic for Bird-HP.

**Conclusion:**

HP should be considered in ILD with greater seasonal changes in serum KL-6 concentrations.

## Background

Krebs von den Lungen-6 (KL-6) is a high molecular weight sialylated MUC1 glycoprotein expressed on type II pneumocytes and bronchial epithelia. In particular, regenerating type II pneumocytes in patients with interstitial lung diseases (ILD) highly express KL-6 [1]. KL-6 may increase in sera after enhanced permeability following destruction of alveolar-capillary barriers and increased production of KL-6 by regenerating type II pneumocytes. Thus, serum KL-6 is increased in patients with ILD with alveolar damage and regeneration of type II pneumocytes, but not increased in patients with bacterial pneumonia [2]. Serum concentrations of KL-6 have been reported to serve as a sensitive biomarker for the diagnosis and monitoring of therapeutic responses in various ILD, including idiopathic pulmonary fibrosis (IPF), non-specific interstitial pneumonia (NSIP), collagen vascular disease-associated interstitial pneumonia (CVD-IP), hypersensitivity pneumonitis (HP), some types of drug-induced pneumonitis, pulmonary sarcoidosis, *Pneumocystis jirovecii* pneumonia (PCP), *Cytomegalovirus* (CMV) pneumonia, and radiation pneumonitis [1-8]. Since KL-6 is not a disease-specific marker and is increased in various ILD, the usefulness of KL-6 in the differential diagnosis among ILD is limited. Serum KL-6 concentrations in patients with eosinophilic pneumonia or organizing pneumonia are usually within normal limits at the time of their diagnosis [4], but may be increased to some extent if the diseases are not treated properly. In addition, higher concentrations of serum KL-6 predict a poorer prognosis in patients with acute exacerbation of IPF, drug-induced pneumonitis, and adult respiratory distress syndrome [4,7-9]. We occasionally observed unexpected increases in serum KL-6 concentrations in patients with ILD during their clinical course. Possible causes of unexpected serum KL-6 elevation in patients with ILD include acute exacerbation of ILD, exacerbation of HP by increased antigen exposure, the development of PCP, CMV pneumonia, drug-induced pneumonitis, or adenocarcinoma of lung, breast, pancreas, ovary, colon and liver.

HP is an extrinsic allergic alveolitis caused by type III and type IV hypersensitivity reactions to inhaled organic antigens such as fungi, bacteria, avian antigens, and feather duvets [10,11]. HP is classified into acute, subacute, and chronic forms, although the subacute form might be a variant of acute HP. Acute HP develops in response to immune complex formation, and the chronic form is mediated by Th1 and likely Th17 CD4^+^ T cells [10]. The prevalence and type of HP is highly dependent on geographical, climatic, occupational differences, and genetic susceptibility. In Japan, summer type house-related HP (House-HP) was reported to be most prevalent (about 70%) in acute HP, and about 60% of chronic HP was bird-related HP (Bird-HP) [12,13]. Some case reports documented that serum KL-6 concentrations vary in response to seasonal changes of antigen exposure levels in patients with HP [14]. Serum KL-6 concentrations may not return to normal levels even after chest radiographs demonstrated improvement following corticosteroids treatment, but did gradually decrease in parallel with improved diffusion capacity of the lungs, which reflects the disease activity of alveolitis in HP [15]. However, the seasonal variation in serum KL-6 concentrations in ILD, including HP, has not been determined. We hypothesized that seasonal variation of serum KL-6 concentrations in patients with HP is greater than for the other ILD. The aim of this study was to determine the seasonal changes of serum KL-6 concentrations in various ILD.

## Methods

### Study subjects

Electronic medical records of patients with registered diagnoses of ILD who consulted our university hospital from April 1, 2009 to March 31, 2014 were reviewed by two pulmonologists. Patients with ILD whose serum KL-6 concentrations were measured at least four times over a period of six months and more than once in one season were selected for analysis. We excluded undiagnosed cases with ILD, ILD cases with lung cancer, drug-induced pneumonitis, radiation pneumonitis, PCP, or CMV pneumonia, each of which has been reported to increase serum KL-6 concentrations. The final study population consisted of 96 ILD patients classified as IPF (n = 16), NSIP (n = 16), CVD-IP (n = 33), House-HP (n = 9), Bird-HP (n = 9), and combined pulmonary fibrosis and emphysema (CPFE, n = 13), based on diagnostic criteria for each. This study complied with the Declaration of Helsinki. The Ethical Review Board of the Kochi Medical School, Kochi University, reviewed the study design and protocol. Approval was waived according to the Ethical Guideline for Clinical Research issued by the Ministry of Health, Labour and Welfare, Japan. Informed consent for the usage of data had been obtained from all patients at their consultation and patients who refused were not included in this study.

### Diagnosis of ILD

IPF was clinically diagnosed based on the ATS/ERS/JRS/ALAT guideline using the presence of basal honeycomb shadows on the chest high-resolution computed tomography (HRCT) (all cases), bronchoalveolar lavage (BAL) (n = 5), transbronchial lung biopsy (TBLB) (n = 5) and exclusion of other causes such as NSIP, CVD-IP, HP, and CPFE [16]. Eight patients with NSIP were histologically diagnosed based on the ATS/ERS multidisciplinary consensus classification of IIPs with surgical lung biopsy [17]. Other 8 NSIP patients were clinically diagnosed by the chest HRCT findings compatible with NSIP pattern, increased lymphocytosis in BAL fluid, lack of granuloma in TBLB specimens, and exclusion of IPF, CVD-IP, HP, or CPFE. CVD-IP was diagnosed based on symptoms, laboratory findings of CVD, and interstitial shadows on chest HRCT. HP was diagnosed by combinations of the presence of specific IgG antibodies against pigeon, budgerigar (Phadia AB, Uppsala, Sweden) for Bird-HP or *Trichosporon asahii* for House-HP, spontaneous remission of interstitial shadows after isolation from causative antigens, positive results on an environmental provocation test, bronchoscopic findings including BAL (n = 17), TBLB (n = 16) and/or surgical lung biopsy (n = 8) [10]. House-HP refers to HP positive for specific IgG antibody against *Trichosporon asahii*. BAL samples were obtained by instillation of 50 ml of saline 3 times and differential cell numbers were determined at the diagnosis of HP without treatment. HP was further classified into acute HP (acute and subacute forms) based on acute onset of symptoms and the appearance of mainly ground glass opacities on chest HRCT, or chronic HP based on gradual progression of symptoms and the presence of mainly interstitial fibrotic shadows on chest HRCT. All cases of Bird-HP in the present study were related to the use of feather products. CPFE was diagnosed by the presence of both emphysematous changes in the upper lobes and interstitial fibrotic changes in the lower lobes on chest HRCT, and exclusion of other causes such as CVD-IP, HP, or drug-induced pneumonitis [18]. Two cases of IPF, five cases of NSIP, five cases of CVD-IP, two cases of House-HP, six cases of Bird-HP, and 12 cases of CPFE were carefully observed throughout their clinical courses without treatment due to patients’ refusal of using pirfenidone, corticosteroids or immunosuppressants. Environmental improvement to avoid exposure to causative antigens was instructed to all patients at the time of their final HP diagnosis.

### Measurement of serum KL-6

Serum samples were collected sequentially from patients 4 to 60 times (median 15 times) during their clinical course (median 903 days, range 195–1703 days). The frequency and duration of KL-6 measurement was not different among each group of ILD (Table 1). Serum KL-6 concentrations were measured by the chemiluminescent enzyme immunoassay LUMIPULSE KL-6 using a KL-6 antibody (EIDIA Co., Ltd, JPN). The cut-off value for serum KL-6 was set at 435 U/ml according to the manufacturer’s instructions. The maximum or minimum concentrations of serum KL-6 in each patient’s clinical course was defined as Kmax or Kmin, respectively. Similarly, serum KL-6 concentrations during the summer from June 1 to September 30 was defined as Smax or Smin, and serum KL-6 concentrations during the winter from November 1 to February 28 as Wmax or Wmin. The variation of serum KL-6 concentrations over each patient’s clinical course (Kmax-Kmin or Kmax/Kmin), during the summer (Smax-Smin or Smax/Smin), during the winter (Wmax-Wmin or Wmax/Wmin), and seasonal changes in serum KL-6 concentrations between summer and winter (Smax-Wmax, Smax/Wmax, Smin-Wmin, or Smin/Wmin) were retrospectively analyzed.

**Table 1:** Seasonal variation of serum KL-6 concentrations in various interstitial lung diseases

	**Total**	**IPF**	**NSIP**	**CVD-IP**	**House-HP**	**Bird-HP**	**CPFE**	***P *** **value**
n	96	16	16	33	9	9	13	-
Numbers of BAL/TBLB/SLB	55/50/18	4/4/0	14/13/8	20/17/2	8/7/5	9/9/3	0/0/0	-
Age	68 (11)	71 (14)	71 (12)	64 (10)*	71 (20)	71 (6)	70 (8)	0.007
Sex (Female)	41	3	9	23*	3	3	0#†	<0.001
No treatment	32	2	5	5	2	6*†	12*#†‡	<0.001
Duration of KL-6 measurement	903 (682)	1013 (670)	718 (326)	1097 (629)	569 (385)	605 (564)	819 (750)	0.072
Frequency of KL-6 measurement	15 (17)	13 (20)	16 (9)	22 (23)	17 (12)	11 (6)	9 (10)	0.133
Kmax	1264 (1182)	1620 (891)	1622 (1734)	993 (1002)	2157 (2162)	1761 (2110)	925 (384)#	0.004
Kmin	532 (349)	667 (335)	590 (328)	433 (287)*	451 (627)	489 (382)	483 (263)	0.070
Kmax-Kmin	686 (958)	793 (510)	1140 (1550)	633 (769)	1173 (1249)	1449 (1467)	360 (216)#§	0.001
Kmax/Kmin	2.46 (1.77)	2.09 (1.02)	3.06 (2.84)	2.47 (1.21)	3.98 (4.11)	4.13 (3.09)	1.95 (0.67)§	0.002
Smax	918 (769)	1231 (548)	1155 (1258)	885 (807)	1932 (1910)	847 (639)	770 (294)	0.090
Smin	588 (390)	696 (347)	715 (293)	438 (313)*	451 (615)	594 (549)	503 (290)	0.033
Smax-Smin	345 (580)	291 (856)	416 (1075)	406 (432)	1002 (1310)	236 (176)	174 (292)	0.072
Smax/Smin	1.61 (1.05)	1.49 (1.13)	1.54 (1.58)	1.76 (0.93)	3.68 (3.63)	1.31 (0.46)‡	1.35 (0.66)	0.047
Wmax	1105 (875)	1320 (799)	1148 (646)	878 (763)	649 (1756)	1572 (1008)†	905 (434)	0.012
Wmin	616 (413)	763 (581)	616 (276)	525 (328)*	491 (855)	750 (687)	551 (380)	0.073
Wmax-Wmin	392 (629)	327 (467)	495 (880)	259 (489)	252 (1120)	839 (721)†	342 (173)	0.051
Wmax/Wmin	1.65 (0.78)	1.46 (0.63)	1.80 (0.86)	1.44 (0.72)	1.47 (0.53)	2.78 (2.34)	1.82 (0.69)	0.078
Smax-Wmax	-51 (511)	-116 (581)	5 (632)	-17 (288)	353 (668)	-819 (609)#†‡	-179 (275)§	0.001
Smax/Wmax	0.93 (0.46)	0.88 (0.44)	1.01 (0.57)	0.96 (0.31)	1.43 (2.31)	0.47 (0.21)*#†‡	0.82 (0.30)‡§	<0.001
Smin-Wmin	-50 (181)	-135 (281)	22 (139)	-62 (114)	-42 (310)	-79 (197)	-54 (206)	0.066
Smin/Wmin	0.90 (0.27)	0.82 (0.27)	1.04 (0.21)	0.87 (0.21)	0.89 (0.20)	0.81 (0.31)	0.89 (0.26)	0.099

The maximum and minimum concentrations of serum KL-6 throughout each patient’s clinical course was defined as Kmax and Kmin. Similarly, serum KL-6 concentrations during the summer (June 1 - September 30) were defined as Smax or Smin, and serum KL-6 concentrations during the winter (November 1 – February 28) as Wmax or Wmin. *IPF*, idiopathic pulmonary fibrosis; *NSIP*, non-specific interstitial pneumonia; *CVD-IP*, collagen vascular disease-related interstitial pneumonia; *House-HP*, house-related hypersensitivity pneumonitis; *Bird-HP,* bird-related hypersensitivity pneumonitis; *CPFE*, combined pulmonary fibrosis and emphysema; *BAL,* bronchoalveolar lavage; *TBLB,* transbronchial lung biopsy; *SLB*, surgical lung biopsy. Data are expressed as the median (interquartile range).

*P < 0.0033 vs IPF.

#*P* <0.0033 vs NSIP.

†*P* < 0.0033 vs CVD-IP.

‡*P* <0.0033 vs House-HP.

§*P* <0.0033 vs Bird-HP.

### Statistical analysis

Data are presented as the median (interquartile range). Statistical analysis of data was performed using IBM SPSS Statistics version 19 for Windows (IBM Corp, NY, USA). The comparisons of serum KL-6 concentrations among ILD were tested by the Kruskal-Wallis test. Differences in serum KL-6 concentrations between two groups were evaluated by the two-tailed Mann–Whitney *U*-test. Serum KL-6 concentrations and KL-6 ratios were further analyzed using receiver operating characteristic (ROC) curves to determine the cut-off levels that resulted in the optimal diagnostic accuracy for the diagnosis of HP, House-HP or Bird-HP. Quantitative differences were tested by the Pearson’s chi-square test for goodness of fit or the Fisher’s exact probability test. A *P*-value of < 0.05 was considered statistically significant. The Holm’s sequential Bonferonni correction was used to adjust the levels of significance for multiple comparisons.

## Results

### Patient characteristics

Patients with CVD-IP were significantly younger than patients with IPF (Table 1). Patients with CVD-IP (23 out of 33) and NSIP (9 out of 16) were predominantly female, and all cases with CPFE were male. Four cases with IPF and one case with NSIP had acute exacerbation during their clinical course [842 (527) days], and two cases of IPF and one case of NSIP were deceased.

### Serum KL-6 concentrations in various ILD

**Figure 1:**
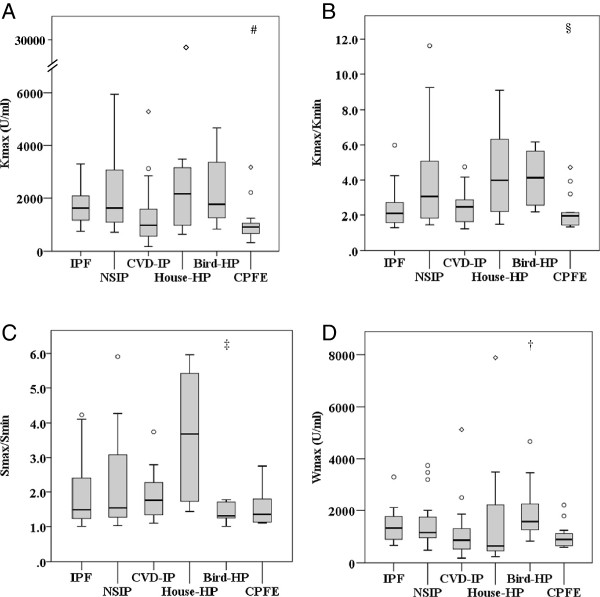
**Serum KL-6 concentrations in various interstitial lung diseases (ILD). (A)** The maximum concentrations of serum KL-6 during their clinical courses (Kmax) in patients with various interstitial lung diseases (ILD). **(B)** The ratio of maximum and minimum concentrations of serum KL-6 (Kmax/Kmin) in various ILD. **(C)** The ratio of maximum and minimum concentrations of serum KL-6 in the summer (Smax/Smin) in various ILD. **(D)** The maximum concentrations of serum KL-6 in the winter (Wmax) in various ILD. IPF, idiopathic pulmonary fibrosis; NSIP, non-specific interstitial pneumonia; CVD-IP, collagen vascular disease-related interstitial pneumonia; House-HP, house-related hypersensitivity pneumonitis, Bird-HP; bird-related hypersensitivity pneumonitis, CPFE; combined pulmonary fibrosis and emphysema. *P < 0.0033 vs IPF, #*P* < 0.0033 vs NSIP, †*P* < 0.0033 vs CVD-IP, ‡*P* < 0.0033 vs House-HP, §*P* < 0.0033 vs Bird-HP.

The maximum KL-6 concentrations over the patients’ clinical courses (Kmax) were significantly higher in patients with NSIP compared to CPFE (Table 1, Figure 1A). The minimum KL-6 (Kmin) was significantly higher in patients with IPF than with CVD-IP. The variation of serum KL-6 concentrations over their clinical courses (Kmax-Kmin or Kmax/Kmin) was significantly greater in patients with Bird-HP than with CPFE (Table 1, Figure 1B). The variation of KL-6 during the summer (Smax/Smin) in patients with House-HP was significantly greater than Bird-HP (*P* = 0.003) and showed a tendency to be greater than CPFE (*P* = 0.004), CVD-IP (*P* = 0.021), IPF (*P* = 0.033), or NSIP (*P* = 0.037) (Table 1, Figure 1C). The maximum KL-6 concentrations during the winter (Wmax) and Wmax-Wmin were significantly higher in patients with Bird-HP than CVD-IP (Table 1, Figure 1D).

### Seasonal variation of serum KL-6 concentrations in different ILD

**Figure 2:**
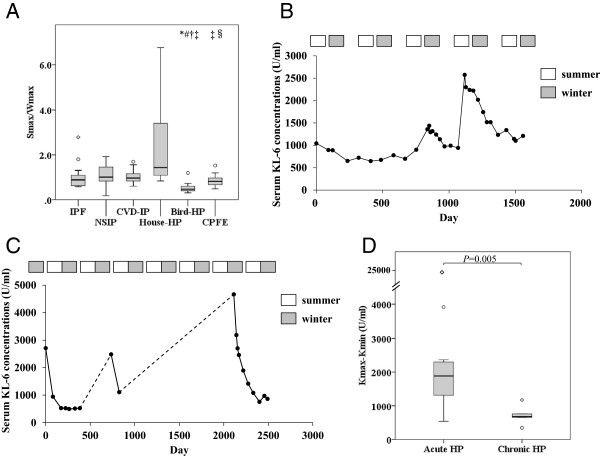
**Seasonal variation of serum KL-6 concentrations in various ILD. (A)** The ratio of maximum concentrations of summer and winter serum KL-6 concentrations (Smax/Wmax) in various ILD. Seasonal changes in serum KL-6 concentrations in a patient with House-HP who had a specific IgG antibody against *Trichosporon asahii***(B)** and in a patient with Bird-HP related to feather pillow use **(C)**. The duration when the patient did not refer to the hospital was shown by dot line in Figure 2C. Corticosteroids were not administered to either patient because of patients’ refusal. The open and closed squares over the figure indicate the summer and winter seasons, respectively. **(D)** Variations of maximal and minimal serum KL-6 concentrations (Kmax-Kmin) in patients with acute HP and chronic HP.

It is particularly noteworthy that Bird-HP and House-HP showed significantly greater seasonal changes in serum KL-6 concentrations than the other ILD. Serum KL-6 concentrations between the summer and the winter (Smax-Wmax or Smax/Wmax) were greater in patients with House-HP than Bird-HP (*P* < 0.001) or CPFE (*P* = 0.003) (Table 1, Figure 2A). Smax/Wmax in patients with Hosue-HP showed a tendency to be greater than IPF (*P* = 0.008) or CVD-IP (*P* = 0.01). Eight out of nine patients (88.9%) with Bird-HP showed greater increases in serum KL-6 concentrations in the winter compared to the summer; as such, Smax-Wmax in patients with Bird-HP showed negative values and Smax-Wmax or Smax/Wmax was significantly lower than the other ILD (Table 1, Figure 2A). These data suggest that serum KL-6 concentrations were significantly increased in the summer in patients with House-HP or in the winter in patients with Bird-HP. Typical examples of sequential seasonal changes in serum KL-6 concentrations in a patient with House-HP who had a specific IgG antibody against *Trichosporon asahii* and in a patient with Bird-HP related to the usage of a feather pillow are shown in Figure 2B and C, respectively. Corticosteroids were not administered to these patients because of patients’ refusal. Serum KL-6 concentrations in the patient with House-HP were significantly increased in the summer season and gradually decreased after environmental improvement and cleaning her house of fungi, but increased again in the next year when fungi regrew (Figure 2B). Serum KL-6 concentrations in the patient with Bird-HP were significantly increased in the winter but gradually decreased in the summer. She ceased coming to the hospital twice because her symptoms had improved naturally. She never used feather duvets, but a feather pillow that she used during a nap in the winter was finally proven to be the cause (Figure 2C).

### Variation of serum KL-6 concentrations in acute or chronic HP

In general, serum KL-6 concentrations are thought to be higher in acute HP compared to chronic HP. Kmax in patients with acute HP (n = 12) was significantly higher than that in chronic HP (n = 6) [2635 (1678) for acute HP vs 1036 (402) for chronic HP, *P* = 0.024]. Kmax-Kmin was also significantly higher in patients with acute HP than chronic HP (1883 (883) for acute HP vs 683 (81) for chronic HP, *P* = 0.005, Figure 2D). These results confirm that variations in serum KL-6 concentrations were significantly greater in acute HP than chronic HP. There is no difference in lymphocytosis between acute HP [48.0 (55.1) %] and chronic HP [18.4 (5.6) %], and no correlation between serum KL-6 concentrations at the same day of BAL and lymphocytosis in BAL fluid.

### Diagnostic value of serum KL-6 concentrations and KL-6 ratios in HP

**Figure 3:**
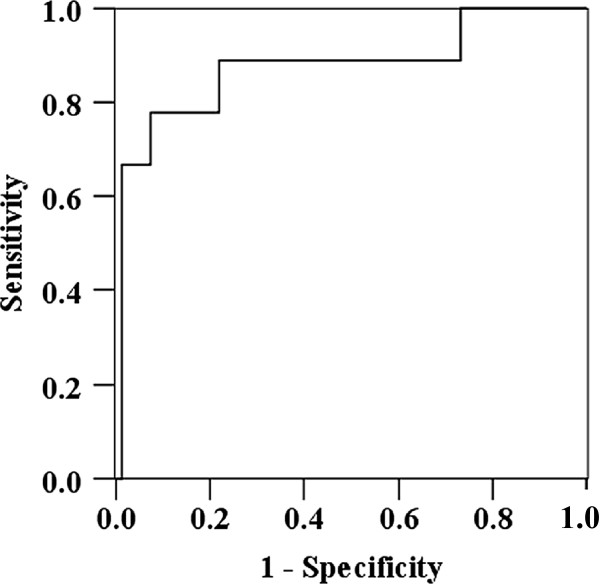
Receiver operating characteristic curve of Smax/Wmax for the diagnosis of Bird-HP.

ROC curves were used to evaluate the diagnostic value of KL-6 concentrations or KL-6 ratios for the diagnosis of HP, House-HP or Bird-HP. Smax/Wmax used for the diagnosis of Bird-HP resulted in the largest area under the curve with 0.878 (Table 2, Figure 3), indicating moderate accuracy. When the Smax/Wmax cut-off level was set at less than 0.61 based on the highest Youden’s index, the sensitivity, specificity, diagnostic accuracy and likelihood ratio for the diagnosis of Bird-HP were 77.8%, 92.7%, 91.2% and 10.63, respectively. These results suggest that seasonal variation of serum KL-6 concentrations is useful for the differential diagnosis of Bird-HP.

**Table 2:** Diagnostic value of KL-6 concentrations and KL-6 ratios for the diagnosis of hypersensitivity pneumonitis (HP), House-HP, and Bird-HP

**Comparison**	**Variables**	**AUC**	***P *** **value**	**Youden’s index**	**Cut-off value**	**Sensitivity (%)**	**Specificity (%)**	**Diagnostic accuracy (%)**	**Likelihood ratio**
HP vs non-HP	Kmax	0.681	0.017	0.316	2532	44.4	87.2	79.2	3.47
	Kmax-Kmin	0.744	0.001	0.427	654	88.9	53.8	60.4	1.93
	Kmax/Kmin	0.757	0.001	0.436	3.15	66.7	76.9	75.0	2.89
House-HP vs non-House-HP	Smax-Wmax	0.774	0.007	0.472	196	66.7	80.5	79.1	3.42
	Smax/Wmax	0.802	0.003	0.461	1.08	77.8	68.3	69.2	2.45
Bird-HP vs non-Bird-HP	Smax-Wmax	0.842	0.001	0.706	-328	88.9	81.7	82.4	4.86
	Smax/Wmax	0.878	<0.001	0.705	0.61	77.8	92.7	91.2	10.63

Cut-off value of Smax-Wmax and Smax/Wmax for the diagnosis of Bird-HP was less than -328 and 0.61, respectively. *AUC*; area under the curve.

## Discussion

We evaluated seasonal variations in serum KL-6 concentrations in various ILD, including IPF, NSIP, CVD-IP, HP, and CPFE, and confirmed that seasonal changes of serum KL-6 concentrations were greater in HP especially Bird-HP than for the other ILD. We also found that variations in serum KL-6 concentrations were significantly greater in acute HP than in chronic HP. Furthermore, seasonal variation of KL-6 concentrations was differentially diagnostic for distinguishing Bird-HP from the other ILD.

Bird-HP and House-HP exhibited increased serum KL-6 concentrations in the winter and summer seasons, respectively. These seasonal differences usually depend on the differences in living environment and patient lifestyles. However, it should be noted that House-HP may be exacerbated not only in the summer but also during the winter season as house heating, increased humidifier usage [19], and improved air tightness of the house may cause mold to spread even in the winter. Also, Bird-HP may be improved even in the winter by removing bird-related products or quit breeding. Therefore, physicians should remain vigilant for possible HP, and detailed history taking is very important to identify causes of increased KL-6 when unexpected increases of KL-6 concentrations are found. Correct, early-stage diagnosis of HP is crucial, since chronic HP can result in a fatal outcome if exposure to the causative antigen is continued [20]. However, differential diagnosis of chronic HP from the other ILD is difficult by only history taking and chest HRCT imaging in clinical settings. Chest HRCT results from chronic HP and IPF patients were similar and difficult to discriminate only by chest HRCT imaging [21,22]. We experienced a case with clinically diagnosed IPF based on chest HRCT findings, histological findings of surgical lung biopsy and detailed history taking but without the usage of feather duvets; the case was finally found to be HP when interstitial shadows were exacerbated after usage of feather duvets by a partner but not by the patient. There is little therapeutic benefit by administering pirfenidone or N-acetyl cysteine to IPF patients [17]. Identifying HP from cases misdiagnosed as IPF is very important because avoidance of the causative antigen is crucial for improving the clinical outcome in HP patients. The best therapy for HP is isolation from causative antigens, improvement of the living environment, and/or systemic administration of corticosteroids for controlling symptoms and stopping progression. The presence of seasonal changes of KL-6 concentrations in each patient, but not comparing KL-6 concentration among patients, is clinically important for suspecting the possibility of HP.

We confirmed that variations in serum KL-6 concentrations were significantly greater in acute than in chronic HP, which may reflect the responses to the different levels of inhaled antigen exposure and the degree of alveolitis. In general, alveolitis with lymphocytosis seems to be less in chronic than acute HP, as lymphocytosis in BAL fluid was less in chronic than acute or subacute HP [23]. However, there is no difference in lymphocytosis between acute HP and chronic HP, and no correlation between serum KL-6 concentrations and lymphocytosis in BAL fluid in the present study, suggesting that increased serum KL-6 concentrations are unlikely to be related to alveolitis with lymphocytosis. Serum KL-6 concentrations are thought to be related to alveolar damage and increased production of KL-6 by regenerating type II pneumocytes. As most cases with acute HP did not undergo surgical lung biopsy, differences in regenerating type II pneumocytes between acute HP and chronic HP were difficult to evaluate. Increased numbers of reactive type II pneumocytes in BAL fluids in patients with HP are reported as similar to PCP and drug-induced pneumonitis [24]. However, differences in regenerating type II pneumocytes between acute HP and chronic HP have not yet been examined.

The limitations of this study include the small number of study subjects, especially for HP, our retrospective approach, lack of seasonal environmental data collection, and the differences in the treatments among the ILD patients, which may affect serum KL-6 concentrations. However, a prospective study to determine the seasonal variation in serum KL-6 concentrations in ILD without treatment is difficult to set up because of ethical issues. Therefore, we decided to use a retrospective study design to address the hypothesis that HP may have greater seasonal serum KL-6 variation than the other ILD. Although small numbers of HP patients were included in the present retrospective study, we clearly demonstrated that Bird-HP and House-HP showed significantly greater seasonal variation in serum KL-6 concentrations compared with the other ILD. Moreover, seasonal variation of serum KL-6 determined by Smax/Wmax less than 0.61 may be useful for the differential diagnosis of Bird-HP. Although we did not collect seasonal environmental data to establish the direct link between seasonal change of KL-6 concentrations and antigen exposure levels, we searched environment of each patients’ house and nearby and found mold spread during summer in the house of patients with House-HP or increased usage of feather products during winter in patients with Bird-HP and therefore speculated that increased antigen exposure could be the cause of seasonal changes in serum KL-6 concentrations.

## Conclusions

Serum KL-6 concentrations exhibited significant seasonal variation presumably in response to seasonal fluctuating antigen exposure levels in patients with HP. Variations in serum KL-6 concentrations were significantly greater in acute HP than in chronic HP. It is necessary to consider the possibility of HP when unexpected seasonal changes in serum KL-6 concentrations are found during the clinical course of ILD.

## Abbreviations

AUC: Area under the curve; BAL: Bronchoalveolar lavage; CMV: Cytomegalovirus; CPFE: Combined pulmonary fibrosis and emphysema; CVD-IP: Collagen vascular disease-associated interstitial pneumonia; HP: Hypersensitivity pneumonitis; HRCT: High resolution computed tomography; ILD: Interstitial lung diseases; IPF: Idiopathic pulmonary fibrosis; KL-6: Krebs von den Lungen-6; NSIP: Non-specific interstitial pneumonia; PCP: *Pneumocystis jirovecii* pneumonia; ROC: Receiver operating characteristic.

## Competing interests

The authors declare that they have no competing interests.

## Authors’ contributions

HO conceived the study, participated in its design and coordination, reviewed medical records, confirmed diagnoses, performed statistical analysis, and drafted the manuscript. SM participated in the selection of patients’ data, reviewed medical records and confirmed diagnoses. SK and TK participated in the selection of patients’ data and made critical suggestions. AY supervised the study and helped draft the manuscript. All authors read and approved the final manuscript.

## Pre-publication history

The pre-publication history for this paper can be accessed here:

http://www.biomedcentral.com/1471-2466/14/129/prepub
